# Transarterial chemoembolization combined with lenvatinib versus transarterial chemoembolization combined with sorafenib for unresectable hepatocellular carcinoma: A systematic review and meta-analysis

**DOI:** 10.3389/fonc.2023.1074793

**Published:** 2023-02-23

**Authors:** Jun-Ning Liu, Ji-Jiang Li, Shu Yan, Guang-Nian Zhang, Peng-Sheng Yi

**Affiliations:** Department of Hepato-Biliary-Pancreas II, Affiliated Hospital of North Sichuan Medical College, Nanchong, China

**Keywords:** hepatocellular carcinoma (HCC), lenvatinib, sorafenib (nexavar), transarterial chemoembolization (TACE), combination therapy

## Abstract

**Background:**

The combination of tyrosine kinase inhibitors (TKIs) and transarterial chemoembolization (TACE) fulfills an important role in the treatment of unresectable hepatocellular carcinoma (uHCC). Among the combination therapies, both lenvatinib and sorafenib combined with TACE are recommended as first-¬line treatments for uHCC. However, at present, limited data are available concerning the efficacy and safety of these two combination therapies in uHCC.

**Methods:**

A detailed systematic search for studies on lenvatinib plus TACE (LEN+TACE) and sorafenib plus TACE (SOR+TACE) was conducted in the online databases PubMed, Embase and The Cochrane Library. The outcome data including overall survival (OS), progression free survival (PFS), time to progression (TTP), tumor response and adverse events (AEs), were independently extracted by two authors in a standardized way.

**Results:**

One randomized controlled trial and five cohort studies with 598 patients (LEN+TACE: 261, SOR+TACE: 337) were included in the meta-analysis. A higher rate of odds ratio (OR) for the objective response rate (ORR) [OR: 3.63; 95% confidence intervals (95% CI): 1.89-6.95; I squared statistic (I2) = 57%, P < 0.001] and disease control rate (DCR) (OR: 3.78; 95% CI: 2.00-7.16; I2 = 52%, P = 0.0001) were observed in the LEN+SOR group compared with the SOR+TACE group. The LEN+TACE group also had significant longer OS [hazard ratio (HR): 0.67; 95% CI: 0.52-0.85; I2 = 1%, P = 0.001], PFS (HR: 0.49; 95% CI: 0.38-0.62; I2 = 0%, P? 0.001) and TTP (HR: 0.62; 95% CI: 0.45-0.84; I2 = 0%, P = 0.002) compared with the SOR+TACE group. The incidence of hypertension (OR: 3.05; 95% CI: 1.45-6.39; P = 0.003) and proteinuria (OR: 5.25; 95% CI: 1.73-15.89; P = 0.003) were significantly higher in the LEN+TACE group than SOR+TACE group, while LEN+TACE group exhibited a lower rate of hand–foot–skin reaction (HFSR) (OR: 0.51; 95% CI: 0.27-0.95; P = 0.03) compared with the SOR+TACE group.

**Conclusion:**

The combination therapy of LEN+TACE showed significant superiority compared with SOR+TACE in terms of its efficacy for patients with uHCC. SOR+TACE should be recommended as a replacement therapy when serious AEs occur during the administration of LEN+TACE as the combination therapy.

## 1 Introduction

Liver cancer is the sixth most common and the third most lethal cancer globally, causing approximately 830,000 deaths in 2020 (1). Hepatocellular carcinoma (HCC), as the dominant type of liver cancer, accounts for 75-85% of all liver cancer-associated deaths (1, 2). Liver surgical resection is still considered as the definitive treatment, with high clinical efficacy for early stage HCC; however, the remaining two-third of patients who do not have early-stage HCC can only receive non-curative treatments (3–5).

Transarterial chemoembolization (TACE) has been widely performed for patients with intermediate-stage HCC. Hepatic arteries, as the most important nutrient arteries for HCC, are selectively blocked by TACE, and the procedure leads to the deposition of high concentrations of chemotherapeutic drugs in the tumors, eventually leading to necrosis of the tumor cells (6). According to the updated Barcelona Clinic Liver Cancer (BCLC) strategy, TACE is still recommended as the main treatment for BCLC stage B patients and partial BCLC stage A patients for whom curative treatment methods are either infeasible or have otherwise failed (7). When these patients are not suitable candidates for TACE, systemic treatment may be considered as the next option.

Tyrosine kinase inhibitors (TKIs) are widely administered for various types of cancer through their ability to specificaly bind the tyrosine kinase domain of endothelial growth factor receptor (EGFR) (8). As a primary treatment method, TKIs have greatly benefited patients with advanced HCC. For example, sorafenib, the first multitargeted TKI for HCC, is able to inhibit tumor growth and angiogenesis through targeting both the Raf/MEK/ERK pathway and receptor tyrosine kinases, thereby significantly improving the median survival rates of patients with advanced HCC (9, 10). In addition, lenvatinib has been shown to suppress vascular endothelial growth factor receptors (VEGFR) 1-3, fibroblast growth factor receptor (FGFR) 1-4, platelet- derived growth factor receptor -alpha (PDGFRα), and proto-oncogenes RET and KIT (11). According to Masatoshi Kudo et al. (12), lenvatinib showed non-inferiority compared with sorafenib with regard to overall survival (OS), and all secondary endpoints indicated statistically significant improvement concerning its use. Another recently study also demonstrated that lenvatinib was associated with prolonged progression free survival (PFS) (13). The emergence of lenvatinib as a therapeutic option, therefore, appears to have challenged the supremacy of sorafenib in the treatment of advanced liver cancer.

With the improvements that are being made in medical knowledge, locoregional therapies combined with systemic therapies for unresectable HCC (uHCC) have been widely accepted, and feature among them are TACE plus TKIs therapies. In addition to sorafenib, other TKIs, including lenvatinib, regarofenib and cabozantinib, have been approved by the United States Food and Drug Administration (FDA) for the treatment of uHCC (14), and so a wide range of suitable combination treatment options are now available for patients. A comparative retrospective study (15) indicated that the median OS time was significantly longer in a lenvatinib plus TACE (LEN+TACE) treatment group compared with the sorafenib plus TACE (SOR+TACE) group (30.5 vs. 20.5 months; *P* = 0.018).

However, by contrast, another cohort study performed by Lee et al. (16) obtained an outcome that identified that the median OS time of the LEN+TACE group compared with the SOR+TACE group was not statistically significant (8.75 vs. 7.57 months; *P* = 0.625). In addition, several related reports have been published to compare the effects between these two therapeutic methods patients with uHCC (17–20).

In order to address this controversial issue and to provide more suitable alternative therapeutic strategies for patients with uHCC, the aim of the present study was to perform a meta-analysis to compare the efficacy and safety of the two combination therapies (i.e., LEN+TACE and SOR+TACE).

## 2 Materials and methods

### 2.1 Search strategy

The present analysis complied with the guidelines specified by The Preferred Reporting Items for Systematic reviews and Meta-Analyses (PRISMA) (21). The available literature was searched using the online databases PubMed, Embase and The Cochrane Library (**Supplementary Data 1**). The following search items were used: “liver neoplasms”, “carcinoma, hepatocellular”, “hepatic*”, “hepato*”, “carcinoma*”, “cancer*”, “tumor*”, “chemoembolization, therapeutic”, “chemoemboli*”, “transarterial”, “TACE”, “lenvatinib”, “lenvaxen”, “sorafenib and “nexavar”. There were no language limitations or other restrictions imposed in the search strategy.

### 2.2 Eligibility criteria

Studies were included if they fulfilled the following criteria: i) the study population comprised patients diagnosed with uHCC; ii) patients with HCC had received LEN+TACE compared with SOR+TACE; iii) the study in question was a randomized controlled trial (RCT), cohort study or case-control study; and iv) the primary outcomes assessed were OS, PFS or time to progression (TTP).

Studies were excluded if they fulfilled the following criteria: i) the patients had previous or current malignant tumors; ii) the patients received immunotherapy and/or targeted therapy before the combination therapy; iii) the study in question was a review, meta-analysis, conference abstract, letter or case report; and iv) study lacked adequate outcomes data or reported on outcomes of no interest.

### 2.3 Data extraction and quality assessment

The data were independently extracted by two authors (JN Liu and JJ Li) in a standardized way, and any disagreements between them were resolved by discussion with a third author (PS Yi). The data extracted from each study were as follows: i) the first author’s name, year of publication, nationality of study population and study design; ii) the sample size, patient demographics and clinicopathological features; iii) the hazard risk (HR) and corresponding 95% confidence intervals (95% CI) for OS, PFS and TTP; iv) the complete response (CR), partial response (PR), stable disease (SD), progressive disease (PD), objective response rate (ORR) and disease control rate (DCR); and v) the incidence of AEs. If the studies did not events data of interest concerning endpoint events and only Kaplan-Meier (K-M) survival curves were available, Engauge Digitizer version 11.3 was used to read the K-M curves to obtain relevant data.

Assessment of the quality of the included studies was undertaken independently by JN Liu and JJ Li. The Newcastle-Ottawa Scale (NOS) was used to assess cohort studies (22), and the Cochrane risk of bias tool was applied for RCTs (23).

### 2.4 Statistical analysis

Review Manager version 5.3 was used for all statistical analyses. The study outcomes included OS, PFS, TTP, tumor response (ORR, DCR, CR, PR, SD and PD) and AEs. The primary outcomes (OS, PFS and TTP) are presented as the HR and 95%CIs, whereas the log-HR and its variance were pooled with the use of an inverse variance weighted average. The tumor response was evaluated according to modified Response Evaluation Criteria in Solid Tumors (mRECIST) criteria (24).

The ORR was defined as CR and PR, whereas the DCR was defined as the sum of CR, PR and SD. Tumor response and AEs are expressed in terms of OR with 95% CIs. According to Q statistics and the I-squared statistic (I^2^) index, studies with *P* < 0.05 and I^2^ > 50% were considered to have high heterogeneity (25). Galbreath radial plots were used to evaluate the heterogeneity of included studies, and sensitivity analysis was used to test the robustness of the primary outcomes date. *P* < 0.05 was considered to indicate a statistically significant value.

## 3 Result

### 3.1 Study selection

**Figure 1:**
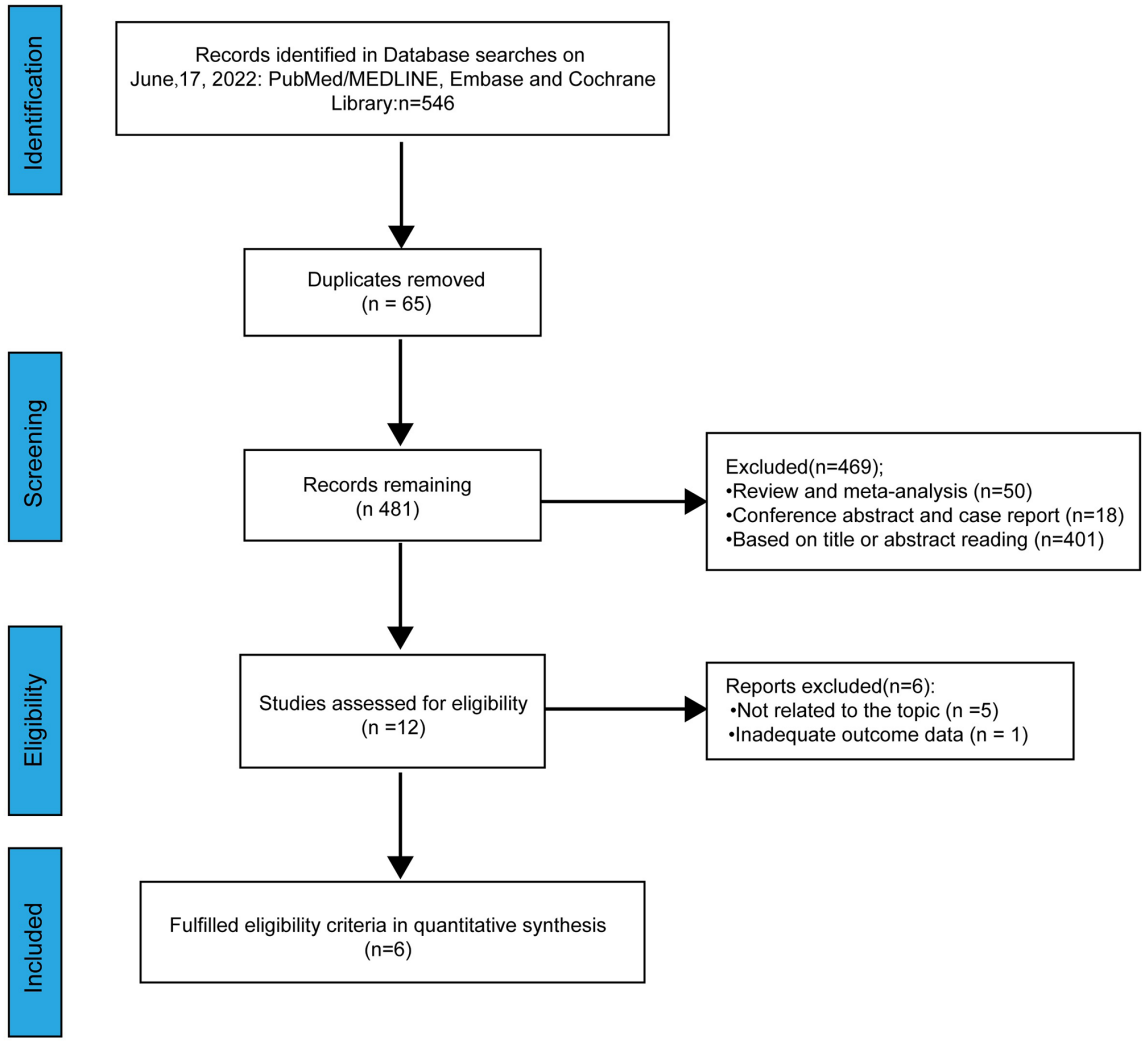
PRISMA flow diagram of the process for the identification of eligible studies.

A total of 546 relevant studies were identified from online databases by the search employed (**Figure 1**). Sixty-five duplicate studies were removed. After screening the title and abstract, assessing the eligibility of the studies and considering other various factors, 475 studies were excluded. Finally, a total of six studies were included in our meta-analysis (15–20).

### 3.2 Study characteristics

The characteristics of the six enrolled studies are presented in **Table 1**. Among them, one study was a single-center prospective RCT from China (17), whereas the other five studies were single-center or multicenter prospective or retrospective cohort studies which were conducted in China, Japan and Korea (15, 16, 18–20). A total of 598 patients with uHCC were included, of which 261 patients were treated with LEN+TACE combination therapy, whereas 337 patients were treated with SOR+TACE. According to these studies, more men than women were included in the analysis. All patients had a class A or B Child–Pugh score, and all patients were identified as being in BCLC B or C stage in the five studies in which the BCLC stage of patients was clearly reported. In addition, the majority of the patients had an Eastern Cooperative Oncology Group Performance Status (ECOG PS) score of 0-1, and were infected with hepatitis B virus (HBV).

**Table 1:** Demographic characteristics of included studies.

Study (Year)	Country	Study design	Treatment	Number of patients	Age (mean/median)	Male/ Female	BCLC stage	Child-Pugh class	ECOG score	Viral hepatitis	AFP (ng/ml)	Tumor number	Largest tumor	Extrahepatic metastasis(Y/N)
Ding 2021	China	RCT	LEN+TACE	32	57 ± 11 ^†^	25/7	All C	A/B: 22/10	0/1: 24/8	HBV/HCV/ other: 30/1/1	23731.4 ± 47862.5^†^	≤3/>3: 27/5	>7/≤7: 25/7	13/19
			SOR+TACE	32	56 ± 11 ^†^	27/5		A/B: 28/4	0/1: 22/10	HBV/HCV/ other: 29/3/0	22862.5 ± 42846.2^†^	≤3/>3: 27/5	>7/≤7: 23/9	9/23
Yang 2021^§^	China	Cohort study	LEN+TACE	38	55.18 ± 10.94	34/4	All C	A/B: 37/1	0/1/2/3: 7/20/11/0	HBV/HCV/ other: 36/0/2	≥400/<400: 24/14	≤3/>3: 22/16	>7/≤7: 24/14	NR
			SOR+TACE	38	54.39 ± 12.17	34/4		A/B: 37/1	0/1/2/3: 3/24/9/2	HBV/HCV/ other: 34/3/1	≥400/<400: 23/15	≤3/>3: 25/13	>7/≤7: 26/12	
Xue 2021 ^§^	China	Cohort study	LEN+TACE	50	54 (49-61)^‡^	46/4	All C	A/B: 41/9	0/1: 37/13	HBV/other: 49/4	1329 (18-10462)^‡^	≤3/>3: 10/40	>5/≤5: 13/37	23/27
			SOR+TACE	100	54 (49-63)^‡^	97/3		A/B: 84/16	0/1: 84/16	HBV/other: 97/3	264 (28-4234)‡	≤3/>3: 24/76	>5/≤5: 19/81	55/45
Shimose 2020	Japan	Cohort study	LEN+TACE	45	75 (45-89)^‡^	40/5	NR	All A	All 0	HBV/HCV/ other: 9/25/11	50.8 (2.8-30772)^‡^	≤3/>3: 6/39	NR	NR
			SOR+TACE	53	73 (54-86)^‡^	46/7				HBV/HCV/ other: 7/34/12	64.5 (1.8-113534)^‡^	≤3/>3: 1/52		
Lee 2020	Korea	Cohort study	LEN+TACE	43	60 (32-85)^‡^	35/8	B/C: 8/35	A/B: 37/6	0/1: 16/27	HBV/HCV/ other: 31/3/9	278.9 (1.4–115807)	NR	NR	24/19
			SOR+TACE	55	63 (43-86)^‡^	42/13	B/C: 8/47	A/B: 52/3	0/1: 22/33	HBV/HCV/ other: 42/2/11	708.8 (1.3–512682)			39/16
Zhang 2022	Chnia	Cohort study	LEN+TACE	53	57.7 ± 11.8 ^†^	44/9	B/C: 27/26	A/B: 52/1	0/1: 43/10	HBV/other: 50/3	≥400/<400: 33/20	≤3/>3: 29/24	>7/≤7: 33/20	17/36
			SOR+TACE	59	58.8 ± 11.1 ^†^	52/7	B/C: 26/33	A/B: 57/2	0/1: 45/14	HBV/other: 55/4	≥400/<400: 34/25	≤3/>3: 33/26	>7/≤7: 37/22	20/39

BCLC, Barcelona Clinic Liver Cancer; ECOG, Eastern Cooperative Oncology Group; AFP, alpha-fetoprotein; HBV, hepatitis B virus; HCV, hepatitis C virus; RCT, randomized controlled trial; LEN, lenvatinib; SOR, sorafenib; TACE, transarterial chemoembolization; Y, yes; N, no. NR, not reported.

^†^Data presented as mean ± SD.

^‡^Data presented as median (range).

^§^Data extraction after propensity score matching method.

For the majority of the included studies, epirubicin was chosen as the chemotherapeutic agent. The embolic agents used included such agents as lipiodol, gelatin sponge or BeadTM. In all studies, the dosage of sorafenib received was 400 mg twice a day, whereas lenvatinib was administered at dose of 8 or 12 mg once a day according to whether the weight of patient was less than or greater than 60 kg. If necessary, the dosage of TKIs were able to be adjusted according to the manufacturer’s protocal (**Table 2**).

**Table 2:** Procedures of lenvatinib plus TACE and sorafenib plus TACE combination therapy.

Study	Year	TACE		Lenvatinib		Sorafenib	Time		
		Chemotherapy	Embolization						
Ding	2021	Epirubicin 50 mg (mixed with lipiodol)	Lipiodol 5-20ml, absorbable Embosphere microspheres 300-500mm	Weight<60 kg, 8 mg qd; weight≥60 kg, 12 mg qd		400 mg bid	Within 3 days before TACE		
Yang	2021	Epirubicin 40-45 mg (mixed with iodized oil)	Iodized oil 2-10 ml, gelatin sponge particle 350-560 µm	Weight<60 kg, 8 mg qd; weight≥60 kg, 12 mg qd		400 mg bid	Within 7 days before or after TACE		
Xue	2021	Doxorubicin 40-80 mg	Emulsion of Callisphere DEB 100-300 µm	Weight<60 kg, 8 mg qd; weight≥60 kg, 12 mg qd		400 mg bid	2-3 weeks prior to the first DEB- TACE session, terminated for 2 days before and after each DEB- TACE session		
Shimose	2020	Epirubicin 20-50 mg or cisplatin 20-50 mg	Lipiodol, 1-mm absorbable gelatin sponge particles	Weight<60 kg, 8 mg qd; weight≥60 kg, 12 mg qd		400 mg bid	After TACE		
Lee	2020	NR	NR	Weight<60 kg, 8 mg qd; weight≥60 kg, 12 mg qd		400 mg bid	After TACE		
Zhang	2022	Lobaplatin 30–50 mg; epirubicin, 10–30 mg	Lipiodol, 300 μm polyvinyl alcohol particles or gelatin sponge particles	Weight<60 kg, 8 mg qd; weight≥60 kg, 12 mg qd		400 mg bid	Initially within 2 weeks after the TACE procedure, an interval of 3–7 days before and after each subsequent TACE session		

TACE, transarterial chemoembolization; DEB, drug-eluting beads.

Only one RCT had been assessed for quality according to the Cochrane risk of bias tool, and this was considered to be a low bias study. In addition, the NOS was used to assess the five cohort studies, which had 7-9 score were therefore deemed to be high quality studies. The details of the quality assessment are shown in **Table 3**.

**Table 3:** Assessment of cohort studies using Newcastle–Ottawa scale.

Study	Year	Selection				Comparability	Exposure/outcome			Total Score
		Represent-ativeness of Cohort★	Selection of control cohort★	Ascertainment of exposure★	Outcome not present at start★	Comparability of cohorts★★	Assessment of outcome★	Length of follow★	Adequacy of follow up★	Total score 9
Lee	2020	★	★	★	★	★	★	★		7
Shimose	2020	★	★	★	★	★	★	★		7
Xue	2021	★	★	★	★	★★	★	★		8
Yang	2021	★	★	★	★	★★	★	★		8
Zhang	2022	★	★	★	★	★	★	★		7

### 3.3 Tumor response

**Figure 2:**
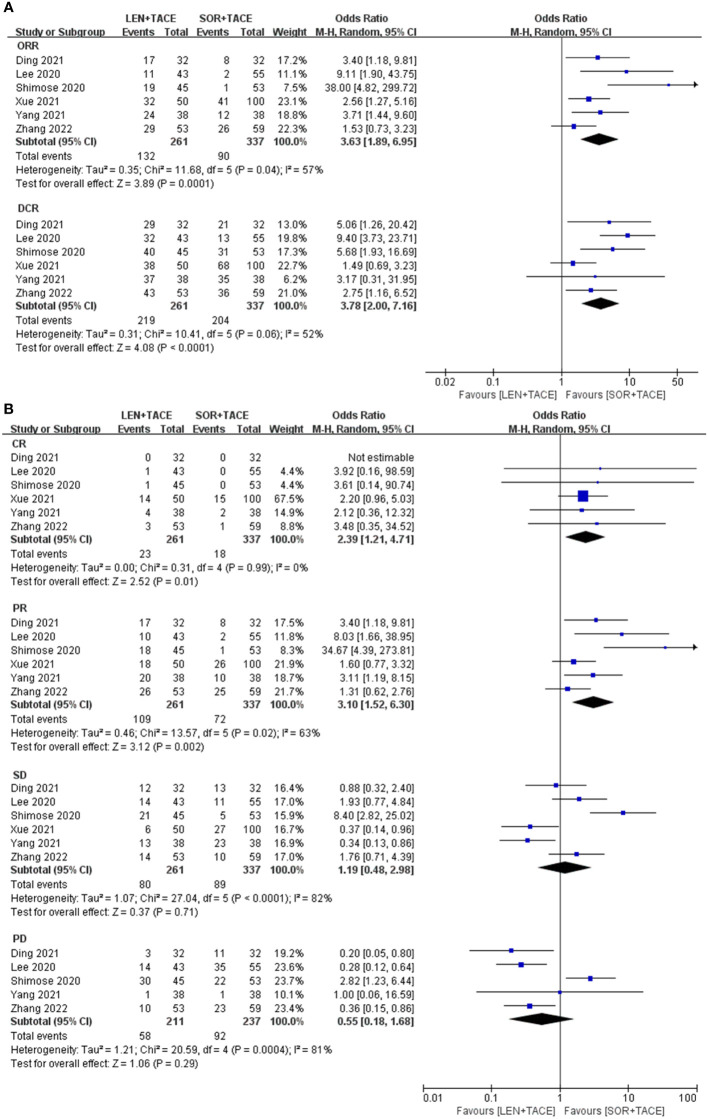
Forest plots for the comparison of **(A)** objective response rate (ORR) and disease control rate (DCR), and **(B)** complete response (CR), partial response (PR), stable disease (SD) and progressive disease (PD).

The tumor response rates, which included ORR, DCR, CR, PR, SD and PD were assessed according to either RECIST or mRECIST criteria (26). A random-effect model was used to pool OR for ORR (OR:3.63; 95% CI: 1.89-6.95; I^2^ = 57%, *P* < 0.001) and DCR (OR: 3.78; 95% CI: 2.00-7.16; I^2^ = 52%, *P* < 0.001), which indicated the significant superiority of the LEN+TACE group over the SOR+TACE group (**Figure 2A**). The higher OR for ORR and DCR in LEN+TACE group might have arisen due to contributions from the rate of CR (OR: 2.39; 95% CI: 1.21-4.71; I^2^ = 0%, *P* = 0.01) and PR (OR: 3.10; 95% CI: 1.52-6.30; I^2^ = 63%, *P* = 0.002). No significant differences, however, were identified in terms of SD (OR: 1.19; 95% CI: 0.48-2.98; I^2^ = 82%, *P* = 0.71) or PD (OR: 0.55; 95% CI: 0.18-1.68; I^2^ = 81%, *P* = 0.29) (**Figure 2B**).

### 3.4 OS, PFS and TTP

**Figure 3:**
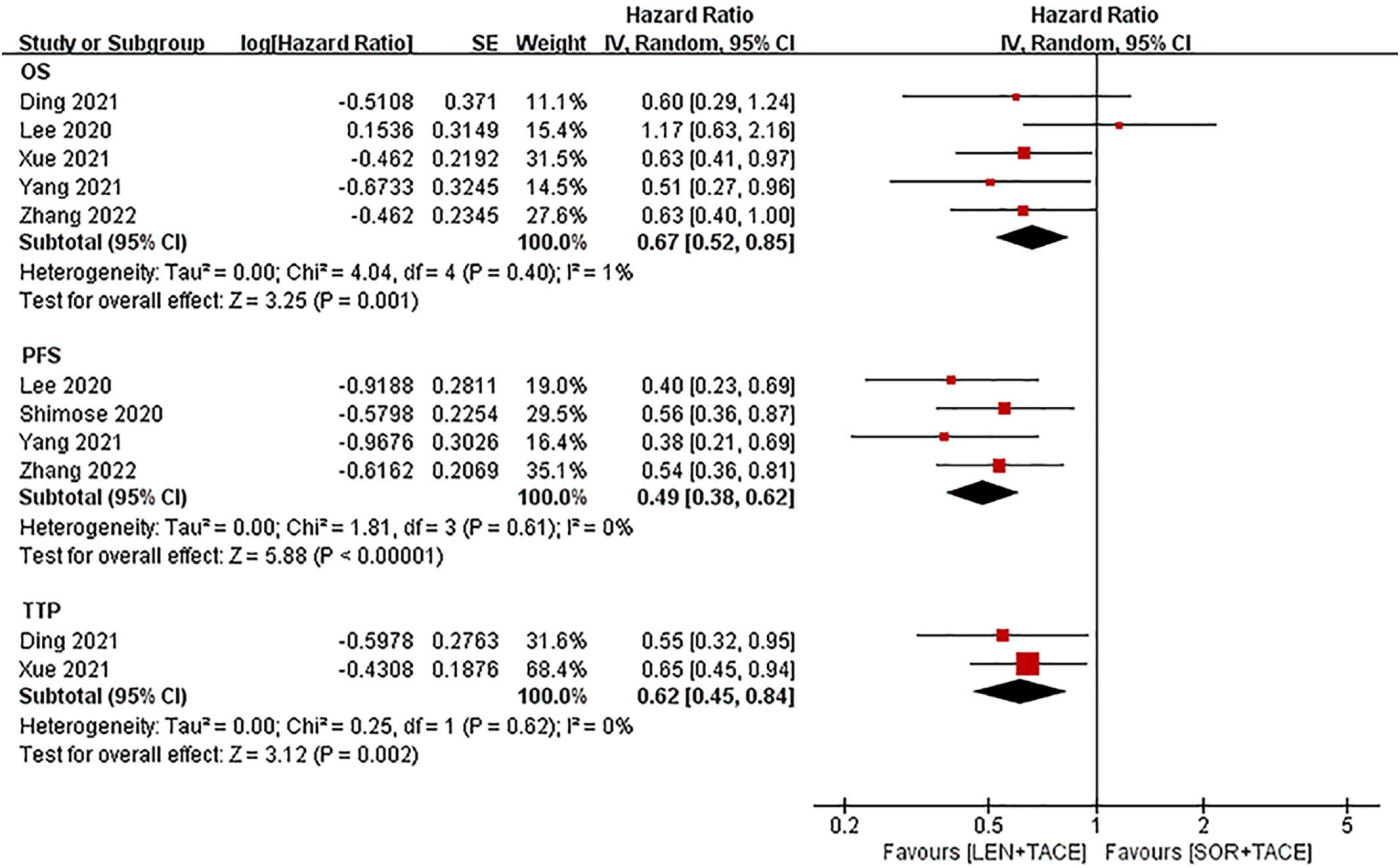
Forest plots for the comparison of overall survival (OS), progression free survival (PFS) and time to progression (TTP).

Five studies reported information on OS; however, the study by Shimose et al. (18) did not provide information on OS since an estimated median OS was not observed in the lenvatinib group. The pooled results illustrated that the LEN+TACE combination therapy group had longer OS rates compared with the SOR+TACE group (HR: 0.67; 95% CI: 0.52-0.85; I^2^ = 1%, *P* = 0.001) (**Figure 3**). After exclusion of the RCT, the pooled OS of the LEN+TACE combination therapy group (HR: 0.68; 95% CI: 0.52- 0.87; I^2^ = 24%, *P* = 0.003) still showed significant superiority over the SOR+TACE group.

The meta-analysis for PFS (HR: 0.49; 95% CI: 0.38-0.62; I^2^ = 0%, *P* < 0.001) showed that the LEN+TACE group was associated with a significantly improved PFS compared with the SOR+TACE group (**Figure 3**).

The TTP value (HR: 0.62; 95% CI: 0.45-0.84; I^2^ = 0%, *P* = 0.002) was reported in only two studies, of which the pooled results showed that the LEN+TACE group had a significantly lower risk of disease progression compared with the SOR+TACE group (**Figure 3**). No heterogeneity in OS, PFS or TTP was observed, so a fixed-effects model was used to pool the effects.

### 3.5 Advertise events

All studies reported AEs, and the 12 most common grade 3/4 AEs from the studies were analyzed. Among them, the most common AEs were pain (14.1%), fever (11.1%) and hypertension (9.2%) for the LEN+TACE combination group, whereas pain (13.1%), fever (12.6%) and HFSR (11.0%) were the most common AEs for SOR+TACE group. The incidence of hypertension (OR: 3.05; 95% CI: 1.45-6.39; *P* = 0.003) and proteinuria (OR: 5.25; 95% CI: 1.73-15.89; *P* = 0.003) were significantly higher in the LEN+TACE group compared with the SOR+TACE group; moreover, the LEN+TACE group showed a lower rate of HFSR (OR: 0.51; 95% CI: 0.27-0.95; *P* = 0.03) compared with SOR+TACE group. Between the two therapy groups, no significant differences were observed in the incidence of diarrhea, transaminitis, decreased appetite, fatigue, nausea and/or vomiting, rash, pain, fever and ascites. The specific details of the pooled analysis of AEs are shown in **Table 4** and **Supplementary Figure 1**.

**Table 4:** Summary of treatment-related adverse events.

Adverse events	LEN+TACE		SOR+TACE		Number of Studies	OR (95% CI)	P value
	Number of events	Rate of events (%)	Number of events	Rate of events (%)			
Hypertension	24	9.2%	10	3.0%	6	3.05 [1.45, 6.39]	0.003
HFSR	14	5.4%	37	11.0%	6	0.51 [0.27, 0.95]	0.03
Diarrhea	12	4.6%	13	3.9%	6	1.08 [0.49, 2.37]	0.85
Transaminitis	12	4.6%	27	8.0%	6	0.54 [0.27, 1.08]	0.08
Decreased appetite	9	4.7%	11	4.1%	4	1.19 [0.48, 2.93]	0.70
Proteinuria	13	7.6%	3	1.3%	4	5.25 [1.73,15.89]	0.003
Fatigue	9	5.0%	13	5.3%	4	1.00 [0.41, 2.45]	1.00
Nausea and/or vomiting	11	6.4%	10	4.4%	4	1.90 [0.78, 4.63]	0.16
Rash	1	0.6%	6	1.7%	4	0.36 [0.06, 2.15]	0.26
Pain	19	14.1%	25	13.1%	3	1.18 [0.62, 2.25]	0.62
Fever	15	11.1%	24	12.6%	3	0.87 [0.44, 1.75]	0.70
Ascites	3	2.4%	4	2.2%	3	1.26 [0.32, 5.00]	0.75

OR, odds ratio; CI, confidence interval; HFSR, hand-foot-skin reaction.

## 4 Discussion

At present, various therapeutic strategies are administered for the treatment of uHCC, among which a combination of TACE and TKIs represents the main treatment. With sorafenib as the first multitargeted TKI for the treatment of HCC, and lenvatinib having been approved by the FDA (14), certain studies comparing lenvatinib and sorafenib monotherapy, or lenvatinib and sorafenib combined with TACE, for uHCC gradually emerged.

The present meta-analysis included five cohort studies and one RCT, comprising total 598 patients, which aimed to explore the efficacy and safety of the LEN+TACE combination therapy group compared with the SOR+TACE therapy group. The pooled analysis demonstrated that, compared with SOR+TACE combination therapy group, the LEN+TACE group exhibited significantly improved OS, PFS and TTP rates. Furthermore, the LEN+TACE group was also revealed to have a higher rate of ORR and DCR. These encouraging results are expected to help us to provide better treatment regimens and to make more accurate clinical decisions.

TACE as the first-line treatment for uHCC effectively causes tumor cells to become necrotic through selectively blocking hepatic arteries and releasing chemotherapeutic drugs (6). However, the side effects present obvious problems, including abnormal liver function and increased expression levels of VEGF and fibroblast growth factor (FGF), thereby promoting angiogenesis, as well as tumor recurrence (27, 28). On this basis, sorafenib and lenvatinib, which are able to inhibit tumor growth and angiogenesis, potentially appeared to be good options to use in combination with TACE for the treatment of uHCC (9–11, 29). A retrospective propensity score-matched analysis reported that the LEN+TACE group appeared to be superior to the TACE group with respect to OS (27.7 vs. 18.4 months; *P* = 0.043), PFS (8.3 vs. 4.6 months; *P* = 0.008) and ORR (64.1 vs. 36.5%; *P* = 0.002) for uHCC; moreover, another retrospective controlled study reached similar conclusions (30, 31). As for the comparison between lenvatinib combined with TACE and lenvatinib monotherapy, a multicenter, open-label, phase III randomized clinical trial in China by Peng et al. (32) demonstrated that the LEN+TACE group had a significantly longer median OS (17.8 vs. 11.5 months; HR = 0.45; *P* < 0.001) and PFS (10.6 vs 6.4 months; HR = 0.43; *P* < 0.001). In addition, a network meta-analysis by Zhang et al. (33), in agreement with above mentioned studies, revealed that the combination therapy of TACE and TKIs was superior to both TACE monotherapy and TKIs monotherapy in terms of its safety and efficacy.

Kudo et al. (12) reported that the median survival time upon administering lenvatinib therapy showed non-inferior compared with sorafenib (13.6 vs. 12.3 months; HR = 0.92, 95% CI: 0.79-1.06). Similarly, a recently published prospective cohort study (34) also found that lenvatinib did not return a survival advantage over sorafenib (HR: 0.82; 95% CI: 0.62- 1.07) for patients with uHCC after inverse probability treatment weighting, although the median survival showed a power of 99% non-inferiority declaration. However, patients being treated with lenvatinib who had previously received TACE experienced a prolonged survival time compared with sorafenib (HR: 0.69; 0.50-0.96), which suggested that the use of lenvatinib appeared to be of greater benefit compared with sorafenib for patients with uHCC who also received TACE. Our meta-analysis compared the two combination therapies of LEN+TACE and SOR+TACE for patients with uHCC. The outcome of a significantly longer OS (HR: 0.67; 95% CI: 0.52-0.85) and PFS (HR: 0.49; 95% CI: 0.38-0.62) in pooled results showed that patients received a greater benefit from a combination of LEN+TACE than they did through treatment with SOR+TACE. Even though only two studies reported TTP values, we still performed a pooled analysis and obtained the same encouraging results. Moreover, we also observed that the LEN+TACE combination treatment group had higher ORR and DCR values possibly due to the higher values for CR and PR compared with the SOR+TACE group, whereas no significant differences in SD and PD were observed comparing between two therapy group. Ding et al. (17) reported the sequential treatment strategies of combination therapy treated patients after disease progression, and patients who received the addition of camrelizumab-based therapies appeared to have better OS than those receiving TKIs monotherapy. However, the best options of sequential treatment after TACE in combination with TKIs need to be found by more large-scale RCTs.

Another meta-analysis by Facciorusso et al. (13) compared lenvatinib vs. sorafenib monotherapies, and also obtained the result of better efficacy using lenvatinib. Based on previous studies and our meta-analysis, the results collectively show excellent efficacy in terms of using LEN+TACE combination treatment compared with both sorafenib monotherapy and SOR+TACE combination therapy.

Regarding the AEs, the 12 most common grade 3/4 AEs were counted in this meta-analysis. Our meta-analysis showed that the LEN+TACE combination therapy group increased the probability of hypertension and proteinuria relative to the SOR+TACE group, although there was a lower risk of HFSR. They were also one of the most common AEs of each of the two groups respectively. Thus, it can be seen that, the safety profiles of sorafenib and lenvatinib were consistent with those reported in the previous study by Kudo et al. (12). Although these events were considered to be common side effects of TKIs, several significant differences were observed the between two groups. This finding would make it possible to the choose an alternative therapeutic strategy whenever serious side effects may occur. Furthermore, pain and fever were also two of the most common identified, although showed no significant differences were identified for these comparing between the two combination therapy groups. Zhang et al. (35) also observed similar AEs, irrespective of whether or not sorafenib monotherapy was compared with SOR+TACE. This result was predominantly due to postembolization syndrome (PES), which is a common complication of TACE and leading to a series of symptoms, including fever, nausea, abdominal pain, vomiting and so on. Fortunately, PES is controllable and previous studies have shown that steroids and Chinese herbal medicine are able to effectively prevent or alleviate it (36–38).

However, this systematic review and meta-analysis did have certain limitations. Firstly, the sample size of included studies was not of a sufficient size that the possibility of overestimated treatment effects could be excluded, and there was only one RCT included out of the total of six included studies, which may have led to a potential risk of selection bias. Secondly, differences did exist in terms of the implementation of the procedures of two therapy groups, and this may have directly affected the outcome of studies. Thirdly, due to the limited number of included studies, it wasn’t possible to perform sensitivity and specific subgroup analysis. Therefore, additional large-scale, multicenter, randomized controlled studies are required.

## 5 Conclusion

In conclusion, the systematic review and meta-analysis performed in the present study have indicated that the combination therapy of lenvatinib and TACE is significantly superior to the use of sorafenib and TACE in terms of its efficacy for patients with uHCC. Sorafenib plus TACE should be recommended as a replacement therapy when serious AEs occur during the use of LEN+TACE combination therapy.

## Data availability statement

The original contributions presented in the study are included in the article/**Supplementary Material**. Further inquiries can be directed to the corresponding author.

## Author contributions

J-NL: design, data acquisition, analysis, interpretation and draft of the manuscript; J-JL: data acquisition, statistics, analysis and draft of the manuscript; SY and G-NZ: data acquisition, software and formal analysis; P-SY: supervision and design of the research. All authors contributed to the article and approved the submitted version.

## References

[1] Sung H Ferlay J Siegel RL Laversanne M Soerjomataram I Jemal A . Global cancer statistics 2020: GLOBOCAN estimates of incidence and mortality worldwide for 36 cancers in 185 countries. CA: Cancer J Clin (2021) 71(3):209–49. doi: 10.3322/caac.21660 33538338

[2] McGlynn KA Petrick JL El-Serag HB . Epidemiology of hepatocellular carcinoma. Hepatol (Baltimore Md) (2021) 73 Suppl 1(Suppl 1):4–13. doi: 10.1002/hep.31288 PMC7577946 32319693

[3] Yang S Lin H Song J . Efficacy and safety of various primary treatment strategies for very early and early hepatocellular carcinoma: A network meta-analysis. Cancer Cell Int (2021) 21(1):681. doi: 10.1186/s12935-021-02365-1 34923980 PMC8684647

[4] Villanueva A . Hepatocellular carcinoma. New Engl J Med (2019) 380(15):1450–62. doi: 10.1056/NEJMra1713263 30970190

[5] Torimura T Iwamoto H . Treatment and the prognosis of hepatocellular carcinoma in Asia. Liver Int (2022) 42(9):2042–54. doi: 10.1111/liv.15130 34894051

[6] Tsurusaki M Murakami T . Surgical and locoregional therapy of HCC: TACE. Liver Cancer (2015) 4(3):165–75. doi: 10.1159/000367739 PMC4608659 26675172

[7] Reig M Forner A Rimola J Ferrer-Fàbrega J Burrel M Garcia-Criado Á . BCLC strategy for prognosis prediction and treatment recommendation: The 2022 update. J Hepatol (2022) 76(3):681–93. doi: 10.1016/j.jhep.2021.11.018 PMC8866082 34801630

[8] Huang L Fu L . Mechanisms of resistance to EGFR tyrosine kinase inhibitors. Acta Pharm Sin B (2015) 5(5):390–401. doi: 10.1016/j.apsb.2015.07.001 26579470 PMC4629442

[9] Abdelgalil AA Alkahtani HM Al-Jenoobi FI . Profiles of drug substances, excipients, and related methodology, Vol. 44. (2019). pp. 239–66. doi: 10.1016/bs.podrm.2018.11.003. 31029219

[10] Llovet JM Ricci S Mazzaferro V Hilgard P Gane E Blanc JF . Sorafenib in advanced hepatocellular carcinoma. New Engl J Med (2008) 359(4):378–90. doi: 10.1056/NEJMoa0708857 18650514

[11] Tohyama O Matsui J Kodama K Hata-Sugi N Kimura T Okamoto K . Antitumor activity of lenvatinib (e7080): An angiogenesis inhibitor that targets multiple receptor tyrosine kinases in preclinical human thyroid cancer models. J Thyroid Res (2014) 2014:638747. doi: 10.1155/2014/638747 25295214 PMC4177084

[12] Kudo M Finn RS Qin S Han KH Ikeda K Piscaglia F . Lenvatinib versus sorafenib in first-line treatment of patients with unresectable hepatocellular carcinoma: a randomised phase 3 non-inferiority trial. Lancet (London England) (2018) 391(10126):1163–73. doi: 10.1016/s0140-6736(18)30207-1 29433850

[13] Facciorusso A Tartaglia N Villani R Serviddio G Ramai D Mohan BP . Lenvatinib versus sorafenib as first-line therapy of advanced hepatocellular carcinoma: a systematic review and meta-analysis. Am J Trans Res (2021) 13(4):2379–87. PMC8129234 34017396

[14] Rimassa L Danesi R Pressiani T Merle P . Management of adverse events associated with tyrosine kinase inhibitors: Improving outcomes for patients with hepatocellular carcinoma. Cancer Treat Rev (2019) 77:20–8. doi: 10.1016/j.ctrv.2019.05.004 31195212

[15] Zhang JX Chen YX Zhou CG Liu J Liu S Shi HB . Transarterial chemoembolization combined with lenvatinib versus transarterial chemoembolization combined with sorafenib for unresectable hepatocellular carcinoma: A comparative retrospective study. Hepatol Res (2022) 52(9):794–803. doi: 10.1111/hepr.13801 35698267

[16] Lee J Sung PS Yang H Lee SK Nam HC Yoo SH . A real-world comparative analysis of lenvatinib and sorafenib as a salvage therapy for transarterial treatments in unresectable HCC. J Clin Med (2020) 9(12). doi: 10.3390/jcm9124121 PMC7767204 33371271

[17] Ding X Sun W Li W Shen Y Guo X Teng Y . Transarterial chemoembolization plus lenvatinib versus transarterial chemoembolization plus sorafenib as first-line treatment for hepatocellular carcinoma with portal vein tumor thrombus: A prospective randomized study. Cancer (2021) 127(20):3782–93. doi: 10.1002/cncr.33677 34237154

[18] Shimose S Kawaguchi T Tanaka M Iwamoto H Miyazaki K Moriyama E . Lenvatinib prolongs the progression-free survival time of patients with intermediate-stage hepatocellular carcinoma refractory to transarterial chemoembolization: A multicenter cohort study using data mining analysis. Oncol Lett (2020) 20(3):2257–65. doi: 10.3892/ol.2020.11758 PMC7400966 32782543

[19] Xue M Wu Y Zhu B Zou X Fan W Li J . Advanced hepatocellular carcinoma treated by transcatheter arterial chemoembolization with drug-eluting beads plus lenvatinib versus sorafenib, a propensity score matching retrospective study. Am J Cancer Res (2021) 11(12):6107–18. PMC8727795 35018245

[20] Yang B Jie L Yang T Chen M Gao Y Zhang T . TACE plus lenvatinib versus TACE plus sorafenib for unresectable hepatocellular carcinoma with portal vein tumor thrombus: A prospective cohort study. Front Oncol (2021) 11:821599. doi: 10.3389/fonc.2021.821599 35004336 PMC8733478

[21] Page MJ McKenzie JE Bossuyt PM Boutron I Hoffmann TC Mulrow CD . The PRISMA 2020 statement: An updated guideline for reporting systematic reviews. BMJ (Clinical Res ed) (2021) 372:n71. doi: 10.1136/bmj.n71 PMC8005924 33782057

[22] Wells GA Shea B O'Connell D Peterson J Welch V Losos M . The Newcastle-Ottawa scale (NOS) for assessing the quality of nonrandomised studies in meta-analyses (2021). Available at: https://www.ohri.ca//programs/clinical_epidemiology/oxford.asp.

[23] Higgins JP Altman DG Gøtzsche PC Jüni P Moher D Oxman AD . The cochrane collaboration's tool for assessing risk of bias in randomised trials. BMJ (Clinical Res ed) (2011) 343:d5928. doi: 10.1136/bmj.d5928 PMC3196245 22008217

[24] Lencioni R Llovet JM . Modified RECIST (mRECIST) assessment for hepatocellular carcinoma. Semin liver Dis (2010) 30(1):52–60. doi: 10.1055/s-0030-1247132 20175033 PMC12268942

[25] Higgins JP Thompson SG Deeks JJ Altman DG . Measuring inconsistency in meta-analyses. BMJ (Clinical Res ed) (2003) 327(7414):557–60. doi: 10.1136/bmj.327.7414.557 PMC192859 12958120

[26] Watanabe H Okada M Kaji Y Satouchi M Sato Y Yamabe Y . New response evaluation criteria in solid tumours-revised RECIST guideline (version 1.1). Gan to kagaku ryoho Cancer chemother (2009) 36(13):2495–501. 20009446

[27] Li X Feng GS Zheng CS Zhuo CK Liu X . Influence of transarterial chemoembolization on angiogenesis and expression of vascular endothelial growth factor and basic fibroblast growth factor in rat with walker-256 transplanted hepatoma: an experimental study. World J Gastroenterol (2003) 9(11):2445–9. doi: 10.3748/wjg.v9.i11.2445 PMC4656518 14606073

[28] Li X Feng GS Zheng CS Zhuo CK Liu X . Expression of plasma vascular endothelial growth factor in patients with hepatocellular carcinoma and effect of transcatheter arterial chemoembolization therapy on plasma vascular endothelial growth factor level. World J Gastroenterol (2004) 10(19):2878–82. doi: 10.3748/wjg.v10.i19.2878 PMC4572123 15334691

[29] Wilhelm S Carter C Lynch M Lowinger T Dumas J Smith RA . Discovery and development of sorafenib: A multikinase inhibitor for treating cancer. Nat Rev Drug Discovery (2006) 5(10):835–44. doi: 10.1038/nrd2130 17016424

[30] Chen YX Zhang JX Zhou CG Liu J Liu S Shi HB . Comparison of the efficacy and safety of transarterial chemoembolization with or without lenvatinib for unresectable hepatocellular carcinoma: A retrospective propensity score-matched analysis. J hepatocellular carcinoma (2022) 9:685–94. doi: 10.2147/jhc.S373250 PMC9354863 35937909

[31] Fu Z Li X Zhong J Chen X Cao K Ding N . Lenvatinib in combination with transarterial chemoembolization for treatment of unresectable hepatocellular carcinoma (uHCC): a retrospective controlled study. Hepatol Int (2021) 15(3):663–75. doi: 10.1007/s12072-021-10184-9 PMC8286947 33877527

[32] Peng Z Fan W Zhu B Wang G Sun J Xiao C . Lenvatinib combined with transarterial chemoembolization as first-line treatment for advanced hepatocellular carcinoma: A phase III, randomized clinical trial (LAUNCH). J Clin Oncol (2022) 41(1):117–27. doi: 10.1200/jco.22.00392 35921605

[33] Zhang Z Wu Y Zheng T Chen X Chen G Chen H . Efficacy of transarterial chemoembolization combined with molecular targeted agents for unresectable hepatocellular carcinoma: A network meta-analysis. Cancers (2022) 14(15). doi: 10.3390/cancers14153710 PMC9367476 35954373

[34] Casadei-Gardini A Scartozzi M Tada T Yoo C Shimose S Masi G . Lenvatinib versus sorafenib in first-line treatment of unresectable hepatocellular carcinoma: An inverse probability of treatment weighting analysis. Liver Int (2021) 41(6):1389–97. doi: 10.1111/liv.14817 33547848

[35] Zhang Y Fan W Wang Y Lu L Fu S Yang J . Sorafenib with and without transarterial chemoembolization for advanced hepatocellular carcinoma with main portal vein tumor thrombosis: A retrospective analysis. oncologist (2015) 20(12):1417–24. doi: 10.1634/theoncologist.2015-0196 PMC4679083 26446238

[36] Yang H Seon J Sung PS Oh JS Lee HL Jang B . Dexamethasone prophylaxis to alleviate postembolization syndrome after transarterial chemoembolization for hepatocellular carcinoma: A randomized, double-blinded, placebo-controlled study. J Vasc interventional Radiol JVIR (2017) 28(11):1503–11.e2. doi: 10.1016/j.jvir.2017.07.021 28941589

[37] Xu H Deng Y Zhou Z Huang Y . Chinese Herbal medicine (Chaihu-huaji decoction) alleviates postembolization syndrome following transcatheter arterial chemoembolization and improves survival in unresectable hepatocellular cancer: A retrospective study. Evidence-Based complementary Altern Med eCAM (2019) 2019:6269518. doi: 10.1155/2019/6269518 PMC6377962 30854013

[38] Xu L Wang S Zhuang L Lin J Chen H Zhu X . Jian pi Li qi decoction alleviated postembolization syndrome following transcatheter arterial chemoembolization for hepatocellular carcinoma: A randomized, double-blind, placebo-controlled trial. Integr Cancer therapies (2016) 15(3):349–57. doi: 10.1177/1534735415617020 PMC5739181 26590124

