# Corrigendum: Transarterial chemoembolization combined with lenvatinib versus transarterial chemoembolization combined with sorafenib for unresectable hepatocellular carcinoma: a systematic review and meta-analysis

**DOI:** 10.3389/fonc.2023.1233247

**Published:** 2023-06-22

**Authors:** Jun-Ning Liu, Ji-Jiang Li, Shu Yan, Guang-Nian Zhang, Peng-Sheng Yi

**Affiliations:** Department of Hepato-Biliary-Pancreas II, Affiliated Hospital of North Sichuan Medical College, Nanchong, China

**Keywords:** hepatocellular carcinoma (HCC), lenvatinib, sorafenib (nexavar), transarterial chemoembolization (TACE), combination therapy

**Error in Figure**

In the published article, there was an error in Figure 2 as published. The two labels “Favours [LEN+TACE]” and “Favours [SOR+TACE]” located on the left and right below the X-axis, should read “Favours [SOR+TACE]” and “Favours [LEN+TACE]” respectively. The corrected Figure 2 and its caption “Forest plots for the comparison of **(A)** objective response rate (ORR) and disease control rate (DCR), and **(B)** complete response (CR), partial response (PR), stable disease (SD) and progressive disease (PD)” appear below.

**Figure 2:**
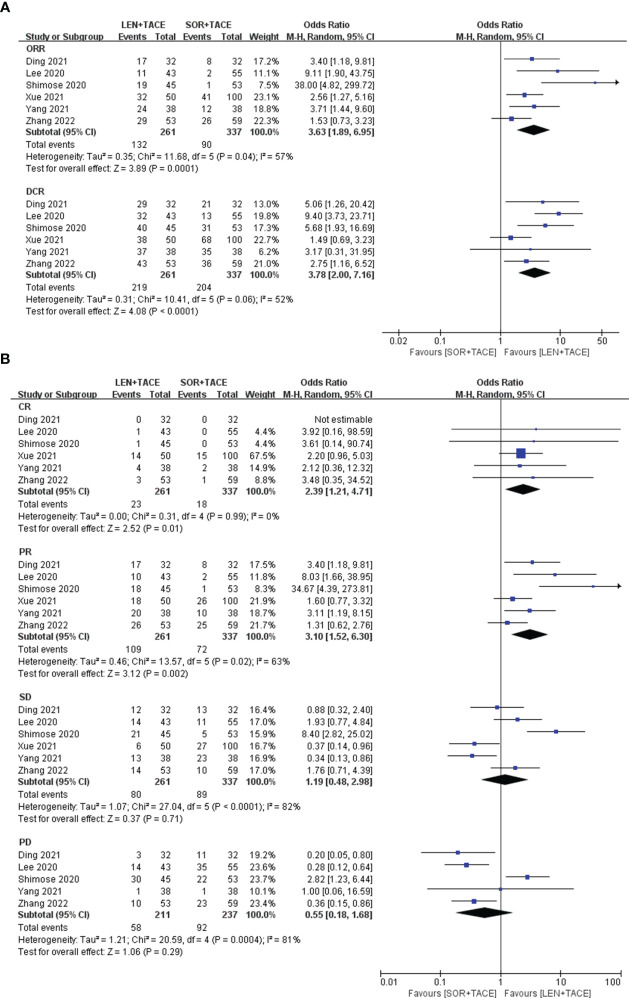
Forest plots for the comparison of **(A)** objective response rate (ORR) and disease control rate (DCR), and **(B)** complete response (CR), partial response (PR), stable disease (SD) and progressive disease (PD).

The authors apologize for this error and state that this does not change the scientific conclusions of the article in any way. The original article has been updated.

