# Supplementary material for: Transarterial chemoembolization combined with lenvatinib versus transarterial chemoembolization combined with sorafenib for unresectable hepatocellular carcinoma: A systematic review and meta-analysis
Source: Front Oncol. 2023 Feb 23;13:1074793. doi: 10.3389/fonc.2023.1074793 (PMC9995877; doi:10.3389/fonc.2023.1074793)
Supplement: Supplementary file 1 [file DataSheet_1.docx]

Supplementary Material

# Supplementary Data

## Supplementary Data 1

**PubMed**

1. "Carcinoma, Hepatocellular"[Mesh] OR "Liver Neoplasms"[Mesh]

2. carcinoma* OR cancer* OR tumor* OR malign* OR neoplasm*

3. Liver* OR hepatic* OR hepato*

4. 1 OR (2 AND 3)

5. "Chemoembolization, Therapeutic"[Mesh] OR Chemoemboli* OR emboli* OR TACE OR TAE OR transarterial OR transcatheter

6. Lenvatinib OR Lenvaxen OR Lenvima

7. Sorafenib OR Nexavar

**Cochrane**

#1 MeSH descriptor: [Carcinoma, Hepatocellular] explode all trees

#2 MeSH descriptor: [Liver Neoplasms] explode all trees

#3 (liver*) OR (hepatic*) OR (hepato*)

#4 (carcinoma*) OR (cancer*) OR (tumor*) OR (malign*) AND (neoplasm*)

#5 #3 AND #4

#6 #1 OR #2 OR #5

#7 MeSH descriptor: [Chemoembolization, Therapeutic] explode all trees

#8 (Chemoemboli*) OR (emboli*) OR (TACE) OR (TAE) OR (transarterial) (Word variations have been searched)

#9 (transcatheter) (Word variations have been searched)

#10 (Lenvatinib) OR (Lenvaxen) OR (Lenvima) (Word variations have been searched)

#11 (sorafenib) OR (Nexavar) (Word variations have been searched)

#12 #7 OR #8 OR #9

#13 #6 AND #10 AND #11 AND #12

**Embase**

1. liver cancer.mp. or exp liver cancer/

2. hepatocellular carcinoma.mp. or exp liver cell carcinoma/

3. (liver* or hepatic or hepato*).mp.

4. (carcinoma* or cancer* or tumor* or malign* or neoplasm*).mp.

5. 1 or 2 or (3 and 4)

6. Chemoembolization.mp. or exp chemoembolization/

7. (Chemoemboli* or emboli* or TACE or TAE or transarterial or transcatheter).mp.

8. 6 or 7

9. Lenvatinib.mp. or exp lenvatinib/

10. (Lenvaxen or Lenvima).mp.

11. sorafenib.mp. or exp sorafenib/

12. Nexavar.mp.

13. 9 or 10

14. 11 or 12

15. 5 and 8 and 13 and 14

# Supplementary Figure

## Supplementary Figure 1

**
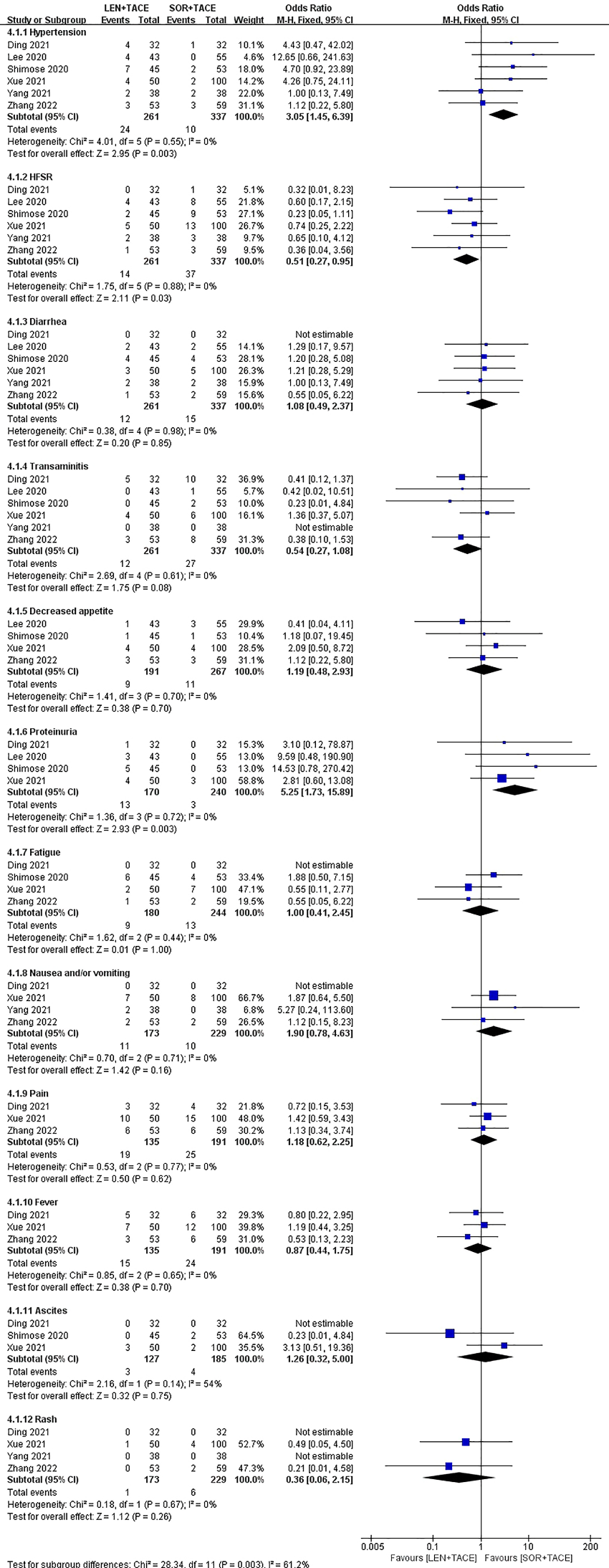
**

**Supplementary Figure 1.** Summary of forest plots of grade 3/4 adverse events. HFSR, hand–foot–skin reaction; TACE, transarterial chemoembolization; LEN, lenvatinib; SOR, sorafenib.
